# Physical Activity and Social Network Use of Adolescents in Overweight and Obesity Treatment

**DOI:** 10.3390/ijerph18136938

**Published:** 2021-06-28

**Authors:** Hagen Wulff, Yanping Duan, Petra Wagner

**Affiliations:** 1 Institute of Exercise and Public Health, University of Leipzig, 04109 Leipzig, Germany; petra.wagner@uni-leipzig.de; 2 Department of Sport Physical Education and Health, Hong Kong Baptist University, Hong Kong, China; duanyp@hkbu.edu.hk

**Keywords:** children and adolescents, obesity and overweight, physical activity, social networks, social media, sociodemographic factors

## Abstract

Tackling obesity among adolescents requires the optimization of existing obesity treatment strategies. For this purpose, social and personal circumstances, individual needs and behavior of therapy participants need to be analyzed to tailor aims, content and methods of therapy interventions to the target groups. A total of 432 obesity therapy participants between 11 and 17 years completed a written survey in a national multi-center study conducted in 2015. The data collection on behavior, in terms of physical activity, media use and sociodemographic variables, was based on questionnaires from the KiGGS, HBSC and JIM studies. The results show that participants were found to be physically active together with friends (75.5%), alone (41.4%) and in sports clubs (34.9%). Girls (OR 1.55) were less likely to participate in sports clubs. Social networks, especially YouTube, WhatsApp, Instagram and Facebook, were widely used. However, differences emerged among sociodemographic groups (e.g., boys vs. girls) regarding the use of social network features. A third of participants reported that smartphone apps regularly encouraged them to exercise. The findings imply that obesity therapy approaches need to be adapted and more differentiated according to the specific needs of the target groups.

## 1. Introduction

Obesity (i.e., pathologically elevated body fat levels compared to total body mass) poses enormous challenges to health services, not just due to its high prevalence [1] but also due to comorbidities [2].

Besides the potentially serious health conditions of the individual, obesity in adults negatively impacts the broader economy, due to lower productivity caused by the incapacity to work combined with the reduced earning capacity [3]. In order to address these challenges, national guidelines are developed to provide obesity treatment strategies for children and adolescents to achieve weight stabilization and reduce obesity-related comorbidity by long-term behavior changes [4]. As lasting behavior changes are difficult to sustain at home, the effectiveness of guideline-based treatment programs is low and requires urgent improvement. National [4] and international [5] publications emphasize this aspect and stress the crucial role of lifestyle for long-lasting therapy effects.

In adolescence, lifestyle (i.e., everyday activities, individual behavior and the behavior of social units) is subject to strong dynamics and influences of social structures, such as domestic environments, peers and friends (peer group) [6]. These dynamics are influenced by sociodemographic factors, e.g., sex, age, education and family background.

Participants with various social backgrounds, personal resources, behavior patterns and needs attend treatment programs. Hence, the programs should be tailored to meet the specific needs of the heterogeneous groups. For this purpose, subgroups consisting of participants with similar health problems and needs can be introduced and suitable therapy components can be developed to maximize the effectiveness and efficiency of treatments.

To develop this specific approach, behavior factors linked to overweight, including media consumption and (insufficient) physical activity [6,7,8,9,10,11], need to be analyzed in relation to determining sociodemographic factors. Particularly in regard to adolescents, media consumption is important, since they spend much of their leisure time using digital media [12]. Social networks play a crucial role as they are accessed daily by nearly all adolescents regardless of their weight [13,14,15].

In line with the uses and gratifications theory [16], media are used for a number of reasons, including self-awareness, entertainment, orientation, information and relaxation. Digital media (especially smartphones and computers) are particularly applied for these purposes as they offer numerous applications (Apps) for recording, processing and transmitting information, such as visualization, audio and video recordings, which meet the users’ needs [17]. In the future, this may affect the use of digital media in therapy programs.

Empirical evidence suggests links between media use, physical activity [18] and the intake of energy-dense foods [8]. High media consumption paired with inactive behavior may lead to reduced metabolism and weight gain in the long term [7]. Similarly, a study by Wulff and Wagner [14] found that high media consumption among obese adolescents attending therapy was related to a lack of exercise. Although these studies provide a starting point for therapy optimization, there is still a need to investigate innovative and more effective technologies, programs and features.

Experience from inpatient and outpatient therapy centers indicates that some therapy participants already use digital media, such as web applications and especially social networks to gain information on health and to motivate themselves to be more active [19]. For instance, GPS tracking apps are used to encourage exercise habits. Apps that record data about nutrition and exercise can be used to calculate the energy balance of users. Before, during and after therapy, digital media can also be used for social interaction [20]. Participants usually inform themselves about therapy centers on websites before starting therapy. This is why therapy centers are now starting to use digital media for therapy and aftercare support [19]. Once therapy is over, participants often use social networks to keep in touch [19]. Therapy centers that use digital media for aftercare are able to cost-effectively fill existing gaps in the current healthcare provision [20,21].

Consequently, there is a need for studies about social and personal resources as well as individual behavior patterns, especially regarding physical behavior and media use of obese adolescents, as respective findings would allow adapting intervention objectives, contents and methods to specific needs of the target groups. This could maximize the effectiveness of current therapy programs. In light of this, the key questions for obesity therapy among adolescents are: How is physical activity related to social circumstances? What social networks are used and to what extent? What features of social networks are used most? Is the use of social networks really related to daily exercise?

## 2. Materials and Methods

In 2015, a national, multi-center cross-sectional analysis was carried out in eight federal states of Germany (Bavaria, Bremen, Hamburg, Hessen, Mecklenburg-West Pomerania, North Rhine-Westphalia, Saxony and Schleswig-Holstein) among 11- to 17-year-old obesity therapy participants. The questionnaire was handed out to the therapy centers, where the therapist personally invited the participants to voluntarily take part in the survey. Participants were only allowed to participate with confirmed parental consent. Participants completed a survey in the first week of their obesity therapy. According to the “Guidelines on treatment of obesity in childhood and adolescence”, all participants had at least a BMI percentile of 90. Out of 530 respondents, 432 participants were included in the study. The remaining 98 adolescents were excluded due to their age (being younger than 11 or older than 17 years).

The data collection on behavior in terms of physical activity, media use and sociodemographic variables, was based on questionnaires from the KiGGS study [22] and the Health Behavior in School-Aged Children (HBSC) World Health Organization Collaborative Cross-National Survey [23], with satisfactory reliability and validity.

For the collection of data of sociodemographic factors, the KiGGS study [24] questionnaire was applied. Data on age, sex, education and employment status of the participants’ parents were collected.

The data collection on physical activity and sports participation was based on the questionnaire of the KiGGS [22] and HBSC studies [23]. The factors measured were: physical activity of a normal week, including outdoor activities, type, volume and setting of performed activities, as well as sport club memberships. Multiple answers were accepted in regard to settings and sport club memberships.

To measure physical activity of the participants, questions from the HBSC questionnaire were used. Based on the WHO recommendations [24] and the question of “how many days the participants were physically active for at least 60 min during the previous week”, participants were split into two groups: “active” (more than 60 min per day) and “inactive” (less than 60 min per day).

To measure the use of social media, questions from the questionnaire of the JIM study were applied [25,26]. Participants were asked about their frequency of social network use (e.g., Facebook, Twitter) and used functions (e.g., texting, view pictures, posting content) based on a five-point scale with the categories: “never”, “at least once a month”, “at least once a week”, “daily” and “more than one hour a day”. To record categories which were not listed, a comment field was provided.

The sample showed the following sociodemographic distribution: 66.0% of all participants were assigned to inpatient obesity centers, while 34.0% were recruited from outpatient centers: 55.8% were female and 44.2% were male. Their median age was 13.2 ± 1.4 (±standard deviation), and 59.5% of the participants were aged 11–13 and 40.5% were 14–17 years old. The participants belonged to different educational institutions, among which 36.5% were from upper middle schools (“Realschule” = middle type of school), 24.9% were from lower middle schools (“Hauptschule” = low type of school), 22.1% were from high schools (“Gymnasium” = high type of school), 3.6% were from schools for children with learning difficulties (“Förderschule”), 1.0% were from vocational colleges (“Berufsschule”) and 0.5% were from primary schools (“Grundschule”). For about 11% of the participants, the school type was unknown. For parents’ employment status, 68.9% of participants had both parents in work, 28.6% had only one parent in work and 2.6% had none in work. Overall, 11.4% of the participants had no siblings, 36.6% had one and 30.8% had two siblings.

To estimate group effects more efficiently, stratified analysis was used to explicitly identify variability-reducing confounders as well as sources that may cause bias. The following strata were used: sex (male, female), age (14–17 and 11–13 years old) and school type (high school, upper and lower middle school). Parents’ employment was divided into: “One or both” in work and “Neither” in work. In terms of physical activity, participants were also classified using the groups “active” or “inactive” by asking them on how many days per “normal” week they exercised for at least one hour. Participants reporting exercise on 0–2 days per week were classified as “inactive” while those who exercised on 5–7 days per week were classified as “active”. “Inactive” groups were used as reference groups (Ref.) for correlation analysis between group membership and the likelihood of increased media consumption or activity. Odds ratios (OR) with a 95% confidence interval (95% CI) were calculated by binary logistic regression. The level of significance was set at 5%.

## 3. Results

### 3.1. Physical Activity

A share of 32.3% of the participants reported that they were exercising together with their families, 34.9% were active in sports clubs, 41.1% exercised by themselves and 75.2% said they exercised with friends. In Table 1, the stratified analysis shows a significant correlation between sociodemographic group membership and the settings “alone” and “club”. Boys were significantly less likely than girls to exercise by themselves (OR 0.45, CI 0.30–0.67) but were 55% more likely to be involved in sports clubs (OR 1.55, CI 1.03–2.31). The results only indicate slight correlations within age groups, as younger adolescents (11–13 years old) showed increased likelihood for club sports activity (OR 1.28, CI 0.85–1.93). Students from lower middle schools (OR 0.73, CI 0.40–0.35) and participants with unemployed parents (OR 0.08, CI 0.01–1.31) were less likely to participate in sports club activities.

Sports club activities were distributed as the following: 17.2% dancing, 15.8% martial arts, 14.0% soccer, 7.7% swimming, 6.7% handball, 4.9% table tennis, 3.9% health sports, 3.5% volunteer work for a fire brigade or the German Red Cross (DRK) and less than 3% other sports. Cycling, soccer, dancing and swimming were usually not practiced in sports clubs. Results show that cycling is the most popular activity, as 69.3% of participants cycled at least once a week. No sociodemographic differences were observed in this regard. Boys (OR 5.81, CI 3.60–9.37) and students from lower middle schools (OR 2.53, CI 1.29–4.99) were significantly more active in football compared to girls, who preferred dancing (OR 0.07, CI 0.04–0.15). A comparison between age groups revealed that younger participants swim significantly more often (OR 1.66, CI 1.03–2.69) and are more likely to play football.

### 3.2. Social Networks

#### 3.2.1. Networks Used Weekly

Participants used several social networks at least once a week. The majority of listed networks were accessed by less than 10% of users, while WhatsApp and YouTube (93.5% each), Instagram (54.7%), Facebook (49.9%), Google+ (38.6%), Skype (35.7%) and Twitter (18.6%) were accessed more frequently by the participants.

The stratified analysis found significant associations between sex and the use of YouTube, Instagram, Facebook and Skype, as well as between age and use of Facebook, school type and use of WhatsApp, Facebook and Google+, and activity status and the use of Skype, see Table 2. No significant correlations were identified regarding parents’ employment status and social network consumption.

#### 3.2.2. Use of Network Features

Among the nine social network features assessed, “write and read messages” (92.2%), “chat” (87.9%) and “watch videos” (87.1%) were used most frequently. Table 3 summarizes the results from the stratified analysis of applied social network features and shows significant links between sex and the use of the following features: “edit my profile”, “read and write messages”, “view other profiles”, “access information” and “play”.

Girls were more likely to use the aforementioned features (apart from “play”). Significant correlations between social network use and age groups mentioned were observed with regard to the features “play” and “access information”. Whilst 11–13-year-olds were more likely to “play” online games within social networks, 14–17-year-olds were more likely to use the same social networks to “access information”. Significant connections between school type and the use of certain network features were analyzed with regard to “play”, “chat”, “post” and “edit my profile”. Students from upper and lower middle schools were more likely to use “chat” and “edit my profile” compared to high school students.

In contrast, students from high schools were less likely to use the features “post” and “play”. Participants with both parents out of work were significantly less likely to use the network features “read and write messages”, “chat”, “view other profiles”, “watch videos” and “access information” compared to participants with both parents in work. No associations between activity levels and the use of certain social network features were found.

#### 3.2.3. Digital Media Used to Encourage Physical Activity

A share of 15.9% of the participants stated that they were encouraged by smartphone apps to exercise at least once a day, 17.8% once a week and 11.8% once a month. In contrast, 54.5% of the participants were not at all encouraged to exercise by smartphone apps. The stratification by sociodemographic groups showed only a significant association with regard to “active” participants being more likely to be encouraged to exercise at least once a week compared to “inactive” participants (OR 2.84, CI 1.55–5.20), see Table 4.

## 4. Discussion

Recent studies indicate that overweight and obese children and adolescents rarely participate in club sports, avoid physical activity in groups and spend much of their leisure time using digital media [15], especially social networks. The results of this study confirm these findings and show that participants primarily exercise alone or together with friends, and considerably less often with family members or in sports clubs. It was found that adolescents preferred physical activity with their peers rather than with their families. This finding can be put into practice by promoting “activity with friends” during therapy, for instance, by encouraging friends to plan and engage in physical activities together.

The results showed that girls, lower middle school students and participants with both parents out of work rarely participate in sports club activities. Obesity therapy participants were found to be less active in sports clubs compared to the KiGGS cohort [9]. An implication of this finding for practice is that therapy participants should be encouraged to join sports clubs. Structures and offerings for target groups need to be improved and used more effectively in the current sports system (especially in rehabilitation, health sports and competitive sports). Thereby, particular attention should be paid to those who are rarely involved in club activities, e.g., girls, students from lower middle schools and adolescents whose parents are unemployed. Additionally, therapy concepts should motivate and enable participants to find the right type of exercise, join sports clubs easily and to attend them regularly, even after therapy.

The health status of obese adolescents is primarily influenced by their behavior and social factors [2,7]. Apart from eating and sleeping habits, exercise and media consumption (especially in social networks) are substantial behavior factors for adolescents aged between 11 and 17 years. This aspect is compounded by the COVID-19 lockdown [8,18,27]. The results of this study support these findings and suggest that participant’s use social networks regularly and extensively, especially WhatsApp, YouTube, Instagram and Facebook. At least 50% of the participants stated that they were active in those networks. The JIM study outlined similar findings, in particular to the question “What are your favorite websites and apps?” [25]. This study contributes to the existing body of literature by offering new additional insights on the social network usage in regard to sociodemographic factors. For instance, YouTube was mainly used by boys, while girls preferred Instagram. Facebook was particularly used by boys, 14–17-year-olds and students attending upper and lower middle schools. The findings underline the importance of social networks for participants in general as well as their varying preferences. The findings can be utilized and implemented in practice by incorporating social networks in future therapy and aftercare approaches, particularly for communication, education and motivation, and to tailor new approaches to the specific needs and preferences of the target groups.

The results of this research show that within different social networks, various network features were used regularly. A high share of participants send and read messages on a daily basis in social networks. This exceeds the findings published in the JIM study [25]. Other frequently used features were “chat”, “watch videos”, “view pictures” and “play”. “Playing games on social network platforms”, for example, is popular among younger participants and students from lower middle schools. Furthermore, girls prefer to spend their time writing messages, editing their profiles and viewing pictures. In line with the JIM and KiGGS study, differences in usage were related to sociodemographic factors. In comparison to the JIM cohort [25], more obesity therapy participants played games on social networks. The findings underline the potential of social network features in future therapy approaches. They imply that it may prove beneficial for treatment providers if they tailor information and treatment materials to the particular needs of the target groups. For instance, sending organizational and therapy-related information in a way participants find most appealing (e.g., in the form of little learning games for younger participants). Another example is the currently used paper-based assignments and homework, which are not always appealing and convenient for young adults, leading to reduced motivation to complete assignments. In contrast, digital material used in social networks may offer an opportunity to overcome this barrier, and allow participants to access assignments anywhere and anytime in a medium suitable to their needs.

An unexpected finding was that the activity status (active, inactive) was not correlated with social network usage. Social networks were used equally by “active” and “inactive” participants. In addition to usage behavior, activating aspects of digital media use were also analyzed. Almost half of the participants were encouraged by smartphone apps to exercise, a third of the participants at least once a week. The analysis of social network use (computer and smartphone) [13] also underlines the enormous extent to which social networks and their features are used, as they play a crucial role in satisfying various personal needs [14,15,16]. Hence, when implementing these findings in practice and developing new therapy approaches, the emphasis should be on teaching obesity treatment participants how to use digital media and social networks effectively and responsibly to improve their physical activity patterns. This is essential to reduce ineffective digital media use and health risks caused by insufficient physical activity.

A limitation of this study is the generalizability of the results, which is limited due to the fact that the sample is not representative, and the media use is constantly changing. Therefore, future studies on physical activity and media use should apply a study design that is representative and timely.

## 5. Conclusions

Despite these limitations, it is concluded that obesity therapy for adolescents should address both physical activity in a social context and digital media activity, particularly in social networks. The findings of this study imply that when developing obesity therapy concepts, aspects such as age, sex and school type should be considered as they are related to the extent and type of digital media use. In the future, it will become increasingly important for therapy providers to incorporate such evidence in their practice and find new ways to utilize digital media and social networks during obesity treatment, in an overall effort to optimize existing therapy results. This is of particular importance as social networks may offer new opportunities to reach obese 11–17-year-olds through their preferred ways of communication. In this regard, the results of this study offer various insights on how to adapt current therapy approaches in accordance with the specific needs of the target groups, such as using social networks for information, education and motivation. Despite the potential of digital media use in obesity therapy, treatment providers also face challenges regarding the use of digital media and social networks. This concerns, for instance, data privacy and security, staff training and cost–benefit analyses. Furthermore, there is still very limited practical guidance on the implementation of digital media in obesity therapy.

Future research needs to address these challenges by further investigating how to design new therapy approaches and achieve therapy objectives by incorporating digital media, social networks and their features in the most effective and efficient manner.

## Figures and Tables

**Table 1:** Indicators and statistics on activity behavior by sociodemographic group.

Physical Activity in Setting		Sex		Age		Type of School			Parents‘ Employment	
	**Total**	**Female**	**Male**	**14–17**	**11–13**	**High**	**Middle**	**Low**	**One or Both**	**Neither**
**Family**										
N (yes)	137/424	78/236	59/188	53/174	84/250	31/87	50/143	33/94	101/293	3/11
In %	32.3	33.1	31.4	30.5	33.6	35.6	35.0	35.1	34.5	27.3
OR		Ref.	0.93	Ref.	1.16	Ref.	0.97	0.98	Ref.	0.71
95% CI			0.62–1.40		0.76–1.75		0.56–1.70	0.53–1.80		0.19–2.75
**Alone**										
N (yes)	176/424	118/236	58/188	80/174	96/250	36/87	69/143	36/94	122/293	5/11
In %	41.5	50.0	30.9	46.0	38.4	41.4	48.3	38.3	41.6	45.5
OR		Ref.	0.45	Ref.	0.73	Ref.	1.32	0.88	Ref.	1.17
95% CI			0.30–0.67		0.49–1.08		0.77–2.26	0.48–1.60		0.35–3.91
**Sports clubs**										
N (yes)	148/424	72/236	76/188	55/174	93/250	34/87	50/143	30/94	106/293	0/11
In %	34.9	30.5	40.4	31.6	37.2	39.1	35.0	31.9	36.2	0.0
OR		Ref.	1.55	Ref.	1.28	Ref.	0.84	0.73	Ref.	0.08
95% CI			1.03–2.31		0.85–1.93		0.48–1.45	0.40–1.35		0.01–1.31
**Friends**										
N (yes)	319/424	170/236	149/188	135/174	184/250	67/87	103/143	72/94	219/293	9/11
In %	75.2	72.0	79.3	77.6	73.6	77.0	72.0	76.6	74.7	81.8
OR		Ref.	1.48	Ref.	0.81	Ref.	0.77	0.98	Ref.	1.52
95% CI			0.94–2.33		0.51–1.27		0.41–1.43	0.49–1.95		0.32–7.20

N (yes): sample size, %: percentage, OR: odds ratio, Ref.: reference group, 95% CI: 95% confidence interval for odds ratios.

**Table 2:** Indicators and statistics on social network use by sociodemographic group.

Social Network		Sex		Age		Type of School			Parents‘ Employment		Physical Activity	
	**Total**	**Female**	**Male**	**14–17**	**11–13**	**High**	**Middle**	**Low**	**One or Both**	**Neither**	**Inactive**	**Active**
**WhatsApp**												
N (yes)	390/417	220/233	170/184	162/171	228/246	74/84	134/139	90/94	275/288	9/9	87/94	127/134
In %	93.5	94.4	92.4	94.7	92.7	88.1	96.4	95.7	95.5	100.0	92.6	94.8
OR		Ref.	0.72	Ref.	0.70	Ref.	3.62	3.04	Ref.	0.93	Ref.	1.46
95% CI			0.33–1.57		0.33–1.61		1.19–10.99	0.92–10.09		0.05–16.85		0.49–4.31
**YouTube**												
N (yes)	373/399	200/220	173/179	149/159	224/240	74/80	125/132	89/94	251/272	9/9	88/92	117/121
In %	93.5	90.9	96.6	93.7	93.3	92.5	94.7	94.7	92.3	100.0	95.7	96.7
OR		Ref.	2.88	Ref.	0.94	Ref.	1.45	1.44	Ref.	1.62	Ref.	1.33
95% CI			1.13–7.34		0.42–2.13		0.47–4.47	0.42–4.92		0.09–28.87		0.32–5.46
**Instagram**												
N (yes)	227/415	146/235	81/180	94/170	133/245	50/85	77/135	55/93	158/285	5/10	50/89	71/135
In %	54.7	62.1	45.0	55.3	54.3	58.8	57.0	59.1	55.4	50.0	56.2	52.6
OR		Ref.	0.50	Ref.	0.96	Ref.	0.93	1.01	Ref.	0.80	Ref.	0.87
95% CI			0.34–0.74		0.65–1.42		0.54–1.61	0.56–1.84		0.23–2.84		0.51–1.48
**Facebook**												
N (yes)	207/415	102/233	105/182	104/171	103/244	34/85	74/137	54/92	147/285	6/11	40/89	66/134
In %	49.9	43.8	57.7	60.8	42.2	40.0	54.0	58.7	51.6	54.5	44.9	49.3
OR		Ref.	1.75	Ref.	0.47	Ref.	1.76	2.13	Ref.	1.13	Ref.	1.19
95% CI			1.18–2.59		0.32–0.70		1.02–3.05	1.17–3.89		0.34–3.78		0.69–2.04
**Google+**												
N (yes)	158/409	90/234	68/175	59/167	99/242	18/84	56/139	45/90	112/284	2/10	28/86	56/134
In %	38.6	38.5	38.9	35.3	40.9	21.4	40.3	50.0	39.4	20.0	32.6	41.8
OR		Ref.	1.02	Ref.	1.27	Ref.	2.47	3.67	Ref.	0.38	Ref.	1.49
95% CI			0.68–1.52		0.84–1.91		1.33–4.61	1.89–7.13		0.08–1.84		0.84–2.62
N (yes)	145/406	69/232	76/174	60/168	85/238	30/85	47/135	34/88	97/279	5/8	23/87	56/134
In %	35.7	29.7	43.7	35.7	35.7	35.3	34.8	38.6	34.8	62.5	26.4	41.8
OR		Ref.	1.83	Ref.	1.00	Ref.	0.98	1.15	Ref.	3.13	Ref.	2.00
95% CI			1.21–2.76		0.66–1.51		0.55–1.73	0.62–2.14		0.73–13.36		1.11–3.59
**Twitter**												
N (yes)	75/404	43/232	32/172	32/167	43/237	20/83	21/136	19/87	48/281	3/9	16/87	23/131
In %	18.6	18.5	18.6	19.2	18.1	24.1	15.4	21.8	17.1	33.3	18.4	17.6
OR		Ref.	1.00	Ref.	0.94	Ref.	0.58	0.88	Ref.	2.43	Ref.	0.95
95% CI			0.61–1.67		0.56–1.55		0.29–1.14	0.43–1.80		0.59–10.04		0.47–1.91

N (yes): sample size, %: percentage, OR: odds ratio, Ref.: reference group, 95% CI: 95% confidence interval for odds ratios.

**Table 3:** Indicators and statistics on social network use by sociodemographic group.

Network Features		Sex		Age		Type of School			Parents‘ Employment		Physical Activity	
	**Total**	**Female**	**Male**	**14–17**	**11–13**	**High**	**Middle**	**Low**	**One or Both**	**Neither**	**Inactive**	**Active**
**Editing my profile**												
N (yes)	189/419	126/236	63/183	74/169	115/250	29/86	68/141	52/93	127/289	5/10	37/89	67/137
In %	45.1	53.4	34.4	43.8	46.0	33.7	48.2	55.9	43.9	50.0	41.6	48.9
OR		Ref.	0.46	Ref.	1.09	Ref.	1.83	2.49	Ref.	1.28	Ref.	1.35
95% CI			0.31–0.68		0.74–1.62		1.05–3.19	1.36–4.57		0.36–4.50		0.79–2.30
**Posting**												
N (yes)	190/409	114/233	76/176	70/166	120/243	33/85	70/137	50/90	131/281	7/10	37/85	68/134
In %	46.5	48.9	43.2	42.2	49.4	38.8	51.1	55.6	46.6	70.0	43.5	50.7
OR		Ref.	0.79	Ref.	1.34	Ref.	1.65	1.97	Ref.	2.67	Ref.	1.34
95% CI			0.54–1.18		0.90–1.99		0.95–2.85	1.08–3.60		0.68–10.54		0.77–2.31
**Writing and reading messages**												
N (yes)	377/409	217/229	160/180	151/163	226/246	77/83	131/139	83/89	267/281	1/9	75/85	124/133
In %	92.2	94.8	88.9	92.6	91.9	92.8	94.2	93.3	95.0	11.1	88.2	93.2
OR		Ref.	0.44	Ref.	0.90	Ref.	1.28	1.08	Ref.	0.01	Ref.	1.84
95% CI			0.21–0.93		0.43–1.89		0.43–3.82	0.33–3.48		0.00–0.06		0.71–4.73
**Chatting**												
N (yes)	362/412	203/228	159/184	147/164	215/248	68/85	129/138	82/89	254/282	2/10	69/85	122/136
In %	87.9	89.0	86.4	89.6	86.7	80.0	93.5	92.1	90.1	20.0	81.2	89.7
OR		Ref.		Ref.	0.75	Ref.	3.58	2.93	Ref.	0.03	Ref.	2.02
95% CI			0.43–1.42		0.40–1.40		1.52–8.47	1.15–7.48		0.01–0.14		0.93–4.39
**Viewing other profiles**												
N (yes)	305/417	192/236	113/181	125/169	180/248	64/86	105/140	72/93	215/288	3/10	68/88	98/137
In %	73.1	81.4	62.4	74.0	72.6	74.4	75.0	77.4	74.7	30.0	77.3	71.5
OR		Ref.	0.38	Ref.	0.93	Ref.	1.03	1.18	Ref.	0.15	Ref.	0.74
95% CI			0.24–0.59		0.60–1.45		0.56–1.91	0.59–2.34		0.04–0.58		0.40–1.38
**Viewing pictures**												
N (yes)	337/410	202/231	135/179	144/168	193/242	73/85	110/135	78/92	237/282	2/9	76/87	111/134
In %	82.2	87.4	75.4	85.7	79.8	85.9	81.5	84.8	84.0	22.2	87.4	82.8
OR		Ref.	0.44	Ref.	0.66	Ref.	0.72	0.92	Ref.	0.05	Ref.	0.70
95% CI			0.26–0.74		0.38–1.12		0.34–1.53	0.40–2.11		0.01–0.27		0.32–1.52
**Watching videos**												
N (yes)	359/412	199/230	160/182	149/169	210/243	74/84	125/139	83/93	245/281	1/10	77/88	116/134
In %	87.1	86.5	87.9	88.2	86.4	88.1	89.9	89.2	87.2	10.0	87.5	86.6
OR		Ref.	1.13	Ref.	0.85	Ref.	1.21	1.12	Ref.	0.02	Ref.	0.92
95% CI			0.63–2.03		0.47–1.55		0.51–2.85	0.44–2.85		0.00–0.13		0.41–2.06
**Playing games**												
N (yes)	273/407	138/228	135/179	97/166	176/241	52/85	89/137	73/90	191/279	5/9	52/84	92/135
In %	67.1	60.5	75.4	58.4	73.0	61.2	65.0	81.1	68.5	55.6	61.9	68.1
OR		Ref.	2.00	Ref.	1.93	Ref.	1.18	2.73	Ref.	0.58	Ref.	1.32
95% CI			1.30–3.08		1.27–2.93		0.67–2.06	1.37–5.40		0.15–2.20		0.75–2.33
**Catching up on the latest information (Events, etc.)**												
N (yes)	231/391	142/221	89/170	106/159	125/232	50/84	88/134	46/85	162/271	0/9	44/80	76/130
In %	59.1	64.3	52.4	66.7	53.9	59.5	65.7	54.1	59.8	0.0	55.0	58.5
OR		Ref.	0.61	Ref.	0.58	Ref.	1.30	0.80	Ref.	0.04	Ref.	1.15
95% CI			0.41–0.92		0.38–0.89		0.74–2.28	0.44–1.48		0.00–0.62		0.66–2.02

N (yes): sample size, %: percentage, OR: odds ratio, Ref.: reference group, 95% CI: 95% confidence interval for odds ratios.

**Table 4:** Indicators and statistics for digital media used to encourage physical activity by sociodemographic group.

Digital Media Used to Encourage Physical Activity												
		**Sex**		**Age**		**Type of School**			**Parents‘ Employment**		**Physical Activity**	
	**Total**	**Female**	**Male**	**14–17**	**11–13**	**High**	**Middle**	**Low**	**One or Both**	**Neither**	**Inactive**	**Active**
**Smartphone Apps**												
N (yes)	142/422	86/234	56/188	67/172	75/250	25/87	49/140	33/94	105/287	4/11	19/93	59/140
In %	33.6	35.8	29.8	39.0	30.0	28.7	35.0	35.1	36.6	36.4	20.4	42.1
OR		Ref.	0.73	Ref.	0.67	Ref.	1.34	1.34	Ref.	0.99	Ref.	2.84
95% CI		0.48–1.10		0.45–1.01		0.75–2.38		0.72–2.52	0.28–3.46			1.55–5.20

N (yes): sample size, %: percentage, OR: odds ratio, Ref.: reference group, 95% CI: 95% confidence interval for odds ratios.

## Data Availability

The data that support the findings of the study are available from the corresponding author upon reasonable request.
